# The Good Food Junction: a Community-Based Food Store Intervention to Address Nutritional Health Inequities

**DOI:** 10.2196/resprot.5303

**Published:** 2016-04-14

**Authors:** Rachel Engler-Stringer, Nazeem Muhajarine, Tracy Ridalls, Sylvia Abonyi, Hassan Vatanparast, Susan Whiting, Ryan Walker

**Affiliations:** ^1^ Community Health and Epidemiology and Saskatchewan Population Health and Evaluation Research Unit University of Saskatchewan Saskatoon, SK Canada; ^2^ Saskatchewan and Population Health and Evaluation Research Unit Saskatoon, SK Canada; ^3^ College of Pharmacy and Nutrition and School of Public Health University of Saskatchewan Saskatoon, SK Canada; ^4^ College of Pharmacy and Nutrition University of Saskatchewan Saskatoon, SK Canada; ^5^ Department of Geography and Planning University of Saskatchewan Saskatoon, SK Canada

**Keywords:** food environments, intervention, natural experiment

## Abstract

**Background:**

This is a 2-year study to assess the early impacts of a new grocery store intervention in a former food desert.

**Objective:**

The purpose of the study is to understand the early health effects of the introduction of a large-scale food and nutrition-focused community-based population health intervention, the Good Food Junction (GFJ) Cooperative Store, in a geographically bounded group of socially disadvantaged neighborhoods (the “core neighborhoods”) in a midsized Canadian city. The GFJ grocery store was tasked with improving the access of residents to healthy, affordable food. The 5 research questions are: (1) What is the awareness and perception of the GFJ store among residents of the core neighborhoods? (2) Are there differences in awareness and perception among those who do and do not shop at the GFJ? (3) Will healthy food purchasing at the GFJ by residents of the core neighborhoods change over time, and what purchases are these individuals making at this store? (4) What early impact(s) will the GFJ have on key health-related outcomes (such as household food security status, vegetable and fruit intake, key aspects of self-reported mental health, self-reported health)? and (5) Are the effects of the intervention seen for specific vulnerable population groups, such as Aboriginal people, seniors (65 years old or older) and new immigrants (settled in Saskatoon for less than 5 years)?

**Methods:**

The research project examined initial impacts of the GFJ on the health of the residents in surrounding neighborhoods through a door-to-door cross-sectional survey of food access and household demographics; an examination of GFJ sales data by location of shoppers' residences; and a 1-year, 3-time-point longitudinal study of self-reported health of GFJ shoppers.

**Results:**

Analyses are on-going, but preliminary results show that shoppers are using the store for its intended purpose, which is to improve access to healthy food in a former food desert.

**Conclusions:**

To our knowledge this is the first large-scale study of a full-service grocery store intervention in a former food desert in Canada that has used multiple data sources, as well as longitudinal analyses, to examine its effects. Its findings will contribute significantly to the knowledge base on food environment interventions.

## Introduction

North American lifestyles generally promote food that is packed with calories (ie, energy-dense food) and offer little incentive for being active [1], particularly in low-income neighborhoods [2]. Specifically, food environments are increasingly recognized as a critical determinant of community and population health [3-5]. Townshend and Lake stated that the food environment “can be broadly conceptualized to include any opportunity to obtain food…. This definition of the food environment can include physical, sociocultural, economic, and policy factors at both micro- and macro-levels” [6].

The presence and accessibility of supermarkets and grocery stores has been linked to improved fruit and vegetable consumption [7], general improvement in healthier food intake [8], and lower body weight [9]. Using national-level data, one US study reported that higher prices for vegetables and fruit are significantly associated with greater gains in BMIs in children between kindergarten and the third grade [10]. There is increasing evidence that poor food environments—such as greater access to fast food and convenience stores and limited access to full-service grocery stores—are more likely to be located in neighborhoods with a lower than average socioeconomic status [9,11-14]. Poor food environments may partly explain why individuals of lower socioeconomic status are more likely to be obese.

While early studies focused primarily on characterizing food environments using geographical and other methods [15], more recent research examined food environment interventions, such as healthy corner store studies [16,17] and the health impacts of opening new supermarkets in “food deserts” (ie, neighborhoods where affordable and nutritious foods are unavailable, requiring residents to travel outside of their neighborhood to access nutritious foods) [18-20]. The body of food environment interventions literature is still very limited in Canada, and results from outside of Canada are contradictory [21-23]. As such, there is a need to conduct larger scale, more systematic, and in particular longitudinal intervention research on food environments.

There have been mixed outcomes in previous research examining food store interventions. Wrigley et al [24] offered the first “before and after” study of a new grocery store development in Leeds, England, in a low-income neighborhood with poor retail access to healthy food. The store was a large-scale chain supermarket. Using fruit and vegetable consumption as a proxy for healthy diet, the authors noted that the introduction of a full-service grocery store significantly improved consumption. Cummins et al [19] also studied the impacts of the opening of a for-profit retail grocery store but showed the intervention had little effect on fruit and vegetable consumption. However, they later argued [25] that the community was not a true food desert and had some access to healthy food. They noted that positive consequences of the grocery store opening included community economic regeneration, increased employment, and a “net reduction in poor psychological health for those who directly engaged with the intervention” [26]. Overall there is uncertainty as to whether grocery store interventions impact healthy eating behavior or other aspects of health.

Drawing on the literature, this study was developed to contribute to the emerging field assessing the impacts of new food access points in low socioeconomic status communities that have previously been characterized as having particularly poor food environments [22,23]. The study is being conducted to assess the early impacts of a new grocery store in a community that had been identified as a food desert [27,28]. The goal of the study is to understand how the introduction of a large-scale food- and nutrition-focused community-based population health intervention (the Good Food Junction Cooperative Store) may impact the health of individuals and families in a geographically bounded group of neighborhoods.

### Intervention and Setting

**Figure 1:**
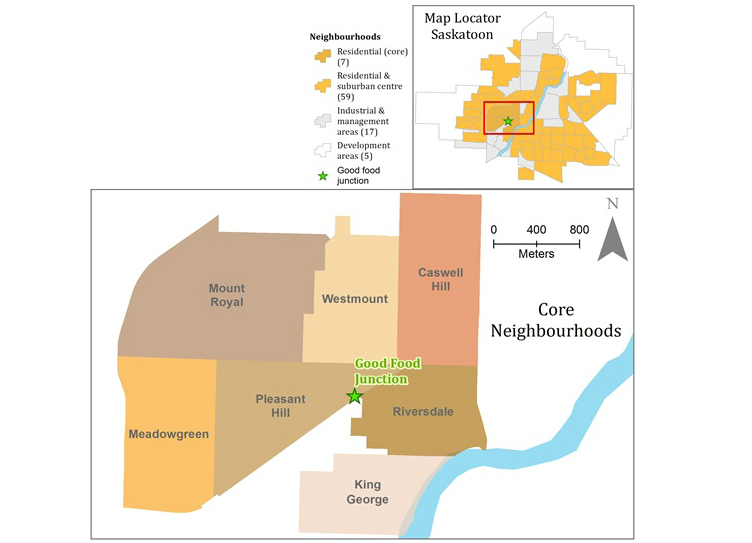
Location of the Good Food Junction Grocery Store.

This study is being conducted in Saskatoon, Saskatchewan, a city of just over 250,000 people. The study is focused on neighborhoods surrounding the Good Food Junction Cooperative grocery store. These are known as the “core neighborhoods,” a set of 7 low socioeconomic status neighborhoods in Saskatoon (Figure 1). The core neighborhoods have several characteristics in common. First, they are located on the west side of the South Saskatchewan River, a river that flows through the center of the city (Figure 1). Second, they are older neighborhoods, built between 1900 and 1930, and finally, they are relatively low-income when compared to the Saskatoon median. These neighborhoods have a relatively high concentration of residents who (1) rent their homes, (2) do not own a vehicle, (3) identify as Aboriginal, (4) are newcomers, or (5) are seniors with fixed incomes [29]. Saskatoon has a large Aboriginal population, compared to other Canadian urban centers, and over half of this population is young (10% of the people living in Saskatoon identify as Aboriginal compared to 3.8% for Canada, and 55% are ≤ 24 y) [30,31]. (The term “Aboriginal” refers to the Indigenous peoples of Canada, descendants of the original inhabitants of this country. It is the legal term used in the Constitution Act of 1982 to refer to First Nations, Métis, and Inuit peoples.)

In September 2012, the Good Food Junction Cooperative Store (GFJ) opened as a result of an 8-year consultation and planning process focused on meeting the food needs of the people living in the area. This grocery store was tasked with improving the access of residents to healthy, affordable food. It is geographically located in the center of a former food desert [27] in the heart of one of the lowest income neighborhoods in the city. The 4900-square-foot store offers a wide range of fresh, frozen, and packaged foods.

The core neighborhoods have had no major chain grocery stores since the mid-1990s, when the last such store closed [28]. The area also saw a loss of small local grocers, going from 12 local grocery stores in 1984 to 5 by 2004 [28]. A 2010 assessment found that residents of the core neighborhoods live much closer to fast food restaurants than to grocery stores, when compared to the citywide average [27].

The GFJ grocery store is housed in a building called Station 20 West [27]. Station 20 West includes the GFJ [32], a community kitchen space, a coffee shop, community meeting space, and several offices for community-based organizations, health services and programming, and community-based research [33].

The goal of this research is to understand and model how the introduction of a large-scale food and nutrition-focused community-based population health intervention (the GFJ) may impact the health of individuals and families in a geographically bounded group of neighborhoods. The study proposal was peer-reviewed and funded by the Canadian Institutes for Health Research, through the Population Health Intervention Research funding stream (grant #127084).

## Methods

The study employed 3 approaches to data collection that roughly correspond to the 5 research questions stated below. There was (1) a cross-sectional door-to-door survey of household food access and demographics (research questions 1 and 2), (2) an analysis of food purchasing sales data obtained directly from the grocery store linked to unique membership numbers (research question 3), and (3) a year-long longitudinal 3-time-point study of food access and self-reported health of GFJ shoppers (research questions 4 and 5). Data collection was conducted from July 2013 through November 2014.

The following research questions informed the study:

1. What is the awareness and perception of the GFJ among residents of the core neighborhood?
2. Are there differences in awareness and perception among those who do and do not shop at the GFJ?
3. Will healthy food purchasing at the GFJ by residents of the core neighborhood change over time, and what purchases are these individuals making at this store?
4. What early impact(s) will the GFJ have on key health-related outcomes (such as household food security status, vegetable and fruit intake, key aspects of self-reported mental health, self-reported health)?
5. Are the effects of the intervention seen for specific vulnerable population groups, such as Aboriginal people, seniors (65 years or older), and new immigrants (settled in Saskatoon for less than 5 years)?

This complex, community-based study of the early impacts of the opening of a full-service grocery store in a former food desert has required a great deal of careful thought, flexibility, and willingness to adapt to the challenges of this type of research with a marginalized and difficult-to-access population. One of the goals of this paper, in addition to describing the research protocol, is to highlight some of the challenges experienced in conducting it and some of the ways in which the study was adapted due to circumstances outside of our control in studying the natural experiment that is the GFJ grocery store.

The project has received ethical approval from the University of Saskatchewan’s Human Behavioural Research Ethics Board (#13-168, approval date: May 30, 2013). Informed consent was obtained from all study participants. Participants have received detailed information outlining study goals and requirements. We have taken due care to inform participants regarding the purpose and the manner in which the data will be collected, used, and secured.

### Cross-sectional Household Food Procurement Survey

In order to answer the first and second research questions, from July of 2013 to November 2013, a cross-sectional survey was administered. A team composed of 4 trained interviewers conducted the door-to-door survey administration in teams of 2. One of the 4 interviewers was a woman who lived adjacent to Station 20 West and was involved in the local community, and the other 3 were women who worked in or were otherwise involved in the community.

The intent of this quantitative survey was to examine the awareness and use of the GFJ grocery store approximately 1 year after opening and other food sources by individuals who lived within walking distance of the store.

With ArcGIS software, a road network of a 750-meter radius (with the GFJ at the center) formed the geographic boundaries for the study. All residential locations within this radius were considered to be within the sample. The distance of 750 meters from the GFJ was chosen to simulate a reasonable walking distance.

Drawing on information from the City of Saskatoon, a total of 1459 residences were considered to be within the 750-meter radius. Of this number, 271 locations were excluded as they were determined to be nonresidential, inaccessible, unsafe, vacant, or nonexistent. We did not want overlap between those who participated in the cross-sectional household food procurement survey and those participating in the longitudinal study described below. Therefore, given that the recruitment period for the 2 components of the research overlapped somewhat, we asked potential participants if they were already participating the longitudinal research. If they answered to the affirmative they were excluded from this component of the research (n=47 participants). The remaining 1141 households were contacted through door-to-door recruitment over a period of 5 months.

Individuals were eligible to participate in the short interviewer-administered survey if they were over 18 years of age, lived in the residence, and were the person primarily responsible for purchasing food for the household.

Topics covered in the survey included whether the participant was aware of the GFJ; if the participant had shopped at the GFJ; which grocery store was their primary grocer; and what mode/s of transportation the participant used to travel to said primary grocery store. Information regarding whether participants accessed a wide range of food programs provided by local nonprofits was also gathered, as well as sociodemographic and household composition information.

Initially, residences within the geographic boundary were randomly sampled for inclusion in the research, but it soon became clear that in order to cover more ground and efficiently collect data, it would be necessary to contact all households within the geographic boundaries of the study area. Interviewers worked in pairs and approached households until the successful completion of a survey, a direct refusal by a resident, or the third unsuccessful attempt to connect with a household member. Appointments were arranged with individuals who wanted to participate but were unable at the initial time of contact. Interviewers left printed door hangers on residences if no one was home, informing residents of the study and ways to participate.

For management of the data collection, the targeted geographical area was divided into 4 quadrants. Visits were systematically recorded on a log sheet, and these sheets were updated before each new round of outreach to potential participants. The schedule for conducting on-the-doorstep outreach was designed to ensure that residences were visited on different days, and at different times. For example, if there had been no one home during a weekday, the following visit was conducted on a weekend or in the early evening.

Of the residences approached, there were 180 refusals and 596 nonresponses after 3 visits. A total of 365 households within the 750-meter radius participated in the survey, which was 32% of all “eligible” households. The average length of interview was 10 minutes. Individuals who participated in the cross-sectional household survey were provided with a $10 gift card to the GFJ as compensation for their time.

All quantitative data for the door-to-door survey was entered and cleaned by study research staff and then analyzed using SPSS version 22 (IBM).

One peer-reviewed paper on this data has been published to date [34] but without description of full methodology as presented here, and full analysis is on-going.

### Multilevel Cross-Sectional Analysis of GFJ Sales Data

In order to answer the third research question, the GFJ shared their sales data to allow the research team to track food sales data by postal code for analysis through a multilevel cross-sectional analytical design.

Since GFJ is a cooperative, members who shop at the store can be tracked to understand their purchasing behavior. For a period of 5 months, from May 1, 2013, to September 30, 2013, the research project paid the membership cost for any shoppers who wished to be a member of the GFJ cooperative. Each GFJ member is assigned a unique membership number, which is recorded at checkout. Food purchasing can be tracked through these membership numbers, as the store records a history of purchases made by each member. Through this database it is possible to analyze the patterns of food purchasing over a period of time.

One limitation with the GFJ sales data is that the membership database contains minimal information (eg, only the members’ postal codes and linked purchasing habits). Individual socioeconomic position is not recorded, but each member’s neighborhood’s socioeconomic position was derived using data from the 2006 Canadian Census, according to Pampalon’s deprivation index [35].

To date, the sales data have been used to observe the food purchasing habits of store members over a 1-year period in order to see whether shoppers living near the GFJ (the “targets” of the intervention) have different shopping patterns compared to GFJ members who live farther away from the GFJ (not targets of the intervention) [36]. As only member-purchases for a year-long period at the GFJ have been analyzed to date (plans for future analyses are described below), these results cannot show the full range of food purchases occurring in the store, nor what members purchased at other stores.

The food purchasing sales data was provided directly by the GFJ grocery store and was entered into a research database, then cleaned and categorized to allow for analysis. Sales data from the GFJ contains information on all food purchases made in the store, along with the membership database.

For our initial analyses, this resulted in information on 72,587 food purchases (by all users) during the period of interest of May 15, 2013, to April 30, 2014. During this period, 526 of the 1109 GFJ members in the data set did not make a purchase, leaving 583 members with 38,190 purchases available for analysis. Of the 583 members with purchase data, 361 (62%) lived in the “core” neighborhoods [36].

Food sales data from the store was categorized into 11 different categories using Stock Keeping Unit codes [37]. The categories were developed in reference to 2 Health Canada tools: Canada’s Food Guide and the Canadian Nutrient File (a database containing information about nutrients in food in Canada) [38,39]. Five categories were considered healthy (eg, fruit, vegetables, meat and alternatives, dairy products, grains) and 6 categories were considered less healthy or nonfood (eg, sugar sweetened beverages, nonnutritive beverages, snack foods, prepared foods, flavoring, nonfood items) [39].

### Longitudinal Food Access and Self-Reported Health Research with Shoppers

In order to answer the fourth and fifth research questions, a year-long 3-time point longitudinal study was conducted to record potential changes in perceived health status in people who made purchases at GFJ. Designed as a food access and health survey, the questions included perceptions of current health status, vegetable and fruit consumption, household food security, and perceptions of a sense of community in their neighborhood, all of which were taken from the Canadian Community Health Survey [40]. In addition, all participants were asked the questions covered in the cross-sectional food access and demographics survey described above.

The health and food access survey was conducted at 3 time points, with a cohort of 156 shoppers at the first time point. The intention was to have the survey administered 3 times with about 6 months between each administration (creating a year time frame of interviews conducted at months 0, 6, and 12). The first time point occurred between July and August 2013, the second was in February through April 2014, and the third administration occurred during July through November 2014.

Participants for this longitudinal study were recruited on-location at GFJ as they entered the store by the same trained interviewers who administered the cross-sectional door-to-door survey. Members of the research team were on-location for 2- to 3-hour periods during the initial recruitment phase. Team members alternated shifts between mornings and afternoons, in an effort to recruit as many shoppers as possible. In order to speed recruitment that was initially proceeding slowly, participants were also recruited through advertisement (predominantly poster and word-of-mouth) in the grocery store and nearby service agencies.

To participate, the respondent had to be over the age of 18, the primary shopper for their household, and was required to have shopped at GFJ at least 3 times in the previous 2 months. To prevent any possible overlap of households, participants were asked whether any other member of their household was already participating in the study. There were no stipulations for participants having to live within a certain geographic distance from the GFJ. It should be noted that residents who participated in the door-to-door survey described above were not recruited to participate in this part of the research.

Every effort was made with individual participants to leave a 6-month period between each iteration of survey administration. The surveys took an average 30 minutes to complete with administration always conducted with a trained interviewer. Due to the need for flexibility, a staff member from Station 20 West was trained to deliver the interview midway through the first iteration of data collection. This individual was well respected and known in the community and was available to participants who could not be accessed by the main interview team. This approach enabled the study to stay connected with participants who were highly transient or did not have phone numbers, and were therefore difficult to contact. Because they regularly attended Station 20 West to connect with resources available there, the staff member was able to ask them if they were willing to conduct follow-up interviews then and there. At each of the 3 data collection time points, participants were compensated for their time with a $25 GFJ gift card.

At the first data collection time point, 156 participants completed the survey. At the second administration of the survey, 129 participants from round 1 participated in the second survey (27 participants were lost to follow-up) but 24 new participants were added (recruitment was done in the same fashion as at the first time point), for a total of 153 participants completing the second round of data collection. In the third administration of the survey, 37 people were lost to follow-up and, therefore, 116 participants completed the survey. A total of 104 participants completed all 3 rounds of data collection and another 37 participants completed 2 rounds for a total of 141 participants with enough data to be included in the analysis.

Participants in this study were also offered a free lifetime membership (usual cost is $5) at the GFJ cooperative. This was done to provide the research team the possibility of analyzing their sales data in conjunction with their survey responses. It is not possible to determine whether being provided with a free membership influenced shopping habits, or whether this action incentivized usage of the store, but it is unlikely given that members do not receive any benefits beyond eligibility to participate in the annual general meeting.

## Results

Data collection for this study is largely complete, but only a small fraction of it has been analyzed to date. Some data from the cross-sectional household food access survey and from sales at the Good Food Junction have been analyzed, but none of the longitudinal self-reported health and food access data from GFJ shoppers have been analyzed to date. The longitudinal research will likely be the largest contribution of this study to the literature, since very little longitudinal research on grocery store interventions in former food deserts has been published.

Two articles have been published to date: Cross-sectional analysis of a community-based cooperative grocery store intervention in Saskatoon, Canada [34], and Examining food purchasing patterns from sales data at a full-service grocery store intervention in a former food desert [36]. From analysis by Lotoski, Engler-Stringer, and Muhajarine of the door-to-door survey, it appears that residents are highly aware of the store (95% of residents were aware of the GFJ at the time of data collection) and most have shopped at the GFJ at least once [34]. Despite this, only 30 of the 251 (12.0%) households surveyed who had ever visited the GFJ used it as their primary grocery store.

Further analysis indicates that compared to residents who did not shop at the GFJ, residents who did shop at the GFJ had lower annual household incomes and were more likely to use local community-based food programs and services in comparison to non-users. This seems to indicate that the GFJ is serving households that are more likely to be facing food insecurity.

From the analysis by Fuller, Engler-Stringer, and Muhajarine [36], it appears that shoppers living in the core neighborhoods are making more healthy food purchases at the GFJ compared to shoppers who live outside of the core neighborhoods. For example, shoppers living in the core neighborhoods spend more on vegetables, and less on meat and alternatives and prepared foods, than shoppers who do not reside in those neighborhoods. This appears to be an indication that people will make healthy food purchases when healthy foods are accessible.

## Discussion

Data analysis is on-going for this study. We will report on our data in several publications. The 3 time point, longitudinal self-reported health and food access research is currently in the early stages of analysis. There are 2 graduate students analyzing the longitudinal self-reported data on health and food access by GFJ shoppers; one focusing on the self-reported health, mental health, and sense of community of GFJ shoppers over time, and the other focusing on vegetable and fruit consumption and household food security of GFJ shoppers over time.

In terms of further analyses to be conducted on our door-to-door food access and demographics survey research, we intend to report on the distances traveled to the primary grocery store of choice and detail the use of community-based food programs of study participants. This will contribute to the literature examining food access practices by residents of low-income neighborhoods. In terms of GFJ sales data, we will also report on the temporality and seasonality of vegetable and fruit and other food purchasing in order to better understand (1) how these issues may impact the sustainability of food environment interventions and (2) how these types of interventions can better respond to the needs of users.

Finally, we are working on a publication that will discuss recruitment and retention challenges and lessons learned in conducting our study. We had to recruit and retain study participants from a marginalized population for this research, particularly very low-income and transient participants, and we found that we learned a great deal from this process, much of which we think will be relevant to others conducting similar research.

### Conclusions

The Good Food Junction Grocery Store opened in 2012 due to a long-term concerted effort by Saskatoon core neighborhood residents and their supporters with the intended purpose of improving access to healthy food in a documented former food desert. Analyses in this study are on-going, but results to date show that shoppers are using the store for its intended purpose [34,36]. Our results show that residents of the neighborhoods directly adjacent to the store have very low household incomes and almost three-quarters of them use anywhere from 1 to 4 community-based food programs, including charitable programs (the food bank was the most widely reported) and numerous others, most of which focus on affordable access to vegetables and fruit. We have also found that the people who shop at GFJ have lower incomes and are more likely to be Aboriginal compared to those who do not. What is clear from our analysis to date is (1) the very low socioeconomic status of our study participants and (2) the importance of better understanding the needs of this population in order to support their access to healthy food.

To our knowledge this is the first large-scale study of a full-service grocery store intervention in a former food desert in Canada that has used multiple data sources, as well as longitudinal analyses, to examine its effects. The study’s findings will contribute significantly to the knowledge base on food environment interventions.

## Appendix

Multimedia Appendix 1 CIHR Peer-Review reports.

## Abbreviations

GFJ: Good Food Junction Cooperative Store
