# Supplementary material for: The Good Food Junction: a Community-Based Food Store Intervention to Address Nutritional Health Inequities
Source: JMIR Res Protoc. 2016 Apr 14;5(2):e52. doi: 10.2196/resprot.5303 (PMC4850278; doi:10.2196/resprot.5303)
Supplement: Multimedia Appendix 1 [file resprot_v5i2e52_app1.pdf]

March/Mars 18, 2013

DC0190GP

Canadian Institutes of Health Research / Instituts de recherche en santé du Canada

Notice of Decision / Avis de décision

Application Number/Numéro de la demande: 297716

Committee Code/Code du comité: IRG

Applicants/Candidats: Dr. Rachel Rosa ENGLER-STRINGER Dr. Nazeem MUHAJARINE

With/Avec: Dr. S. ABONYI Dr. C. NEUDORF Dr. H. VATANPARAST Prof. R. WALKER Dr. S. WHITING

Institution paid/ Établissement payé: University of Saskatchewan

Title/Titre: The Good Food Junction: A Community-Based Food Intervention to Reduce Nutritional Health Inequities

Primary Inst./ Inst. principal: Population and Public Health

Other Related Inst./ Autres inst. connexes: Nutrition, Metabolism and Diabetes

**Competition Outcome/Résultats du concours:**	Operating Grant: Population Health Intervention Research<br>December/Décembre 14, 2012
**Number in competition/Nbre de demandes dans le concours:**	31
**Number approved/Nbre de demandes approuvées:**	17

**Decision on your application/ Décision sur votre demande:**	Approved
**Average annual amount/ Montant annuel moyen:**	The term and amounts can be found on the Authorization for Funding (AFF).
**Equipment amount/ Montant pour les appareils:**	
**Term/Durée:**	

Peer Review Committee Recommendation, for your information and use/ Recommandation du comité d'examen par les pairs, pour fins d'information et d'utilisation:

**Committee/Comité:**	Operating Grant: Population Health Intervention Research
**Application rank within the competition/ Rang de la demande dans ce concours:**	8
**Percent Rank Within the Competition/ Rang en pourcentage au sein du concours:**	25.81%
**Rating/ Cote:**	3.90
**Recommended average annual amount/ Montant annuel moyen recommandé:**	
**Recommended equipment amount/ Montant recommandé pour les appareils:**	

*** Applications receiving a score of less than 3.5 on any evaluation criteria will not be considered for Funding. / Les demandes qui ont reçu une note inférieure à 3.5 pour n'importe quel des critères d'évaluation ne sont pas admissibles.

This document is for information only. Official payment is stated on the CIHR Authorization for Funding.
Document à titre d'information seulement. Le paiement officiel est indiqué sur l'autorisation de financement des IRSC.

CIHR IRSC

Institute of Aboriginal Peoples' Health
Institute of Aging
Institute of Cancer Research
Institute of Circulatory and Respiratory Health
Institute of Gender and Health
Institute of Genetics
Institute of Health Services and Policy Research
Institute of Human Development, Child and Youth Health
Institute of Infection and Immunity
Institute of Musculoskeletal Health and Arthritis
Institute of Neurosciences, Mental Health and Addiction
Institute of Nutrition, Metabolism and Diabetes
Institute of Population and Public Health

Institut de la santé des Autochtones
Institut du vieillissement
Institut du cancer
Institut de la santé circulatoire et respiratoire
Institut de la santé des femmes et des hommes
Institut de génétique
Institut des services et des politiques de la santé
Institut du développement et de la santé des enfants et des adolescents
Institut des maladies infectieuses et immunitaires
Institut de l'appareil locomoteur et de l'arthrite
Institut des neurosciences, de la santé mentale et des toxicomanies
Institut de la nutrition, du métabolisme et du diabète
Institut de la santé publique et des populations

March 18, 2013

Dr. Rachel Engler-Stringer
Community Health and Epidemiology
College of Medicine
University of Saskatchewan
107 Wiggins Road
Saskatoon, Saskatchewan S7N 5E5

Dear Dr. Engler-Stringer,

On behalf of the Canadian Institutes of Health Research (CIHR) Institute of Population and Public Health (IPPH), the Institute of Nutrition, Metabolism and Diabetes (INMD) and the Public Health Agency of Canada – Office of Public Health Practice (PHAC-OPHP), I am pleased to inform you that your recent application for funding entitled *"Informing the "The Good Food Junction: A Community-Based Food Intervention to Reduce Nutritional Health Inequitiest"* was approved for funding through the Operating Grant: Population Health Intervention Research (Fall 2012) program. Your Authorization for Funding will follow in the mail. Please note that while this grant is for 2 years, all funds will be released in Fiscal Year 2012-13.

CIHR requires your consent in order to share copies of your full application and final report with funding partners involved in this funding opportunity. Please contact the coordinator listed below by Wednesday, April 10th, 2013 in order to confirm your consent.

As you are receiving this letter through ResearchNet, you will also find posted your application reviews. As CIHR does not notify the other applicants involved in a given application of the decision taken, we ask that you inform those individuals along with their research institutions (if different from your own) of the outcome of this application.

Please do not contact members of the peer review committee. Should you have any questions about the review process and the sharing of information with funding partners, please contact Lisa Rivet, Program Coordinator, by email at:
lisa.rivet@cihr-irsc.gc.ca.

Congratulations on your success in this competition.

Sincerely,

Allison Jackson
Deputy Director, Strategic Program Design and Analytics
Research and Knowledge Translation

cc: Julie Senécal, CIHR Institute of Population and Public Health
Paul Bélanger, CIHR Institute of Nutrition, Metabolism and Diabetes
Deborah Jordan, Public Health Agency of Canada – Office of Public Health Practice

Canadian Institutes of Health Research
160 Elgin Street, 9th Floor, Address Locator 4809A
Ottawa, Ontario K1A 0W9 Tel: 613-941-2672
Fax: 613-954-1800 **www.cihr-irsc.gc.ca**

Instituts de recherche en santé du Canada
160, rue Elgin, 9e étage, Indice de l'adresse 4809A
Ottawa (Ontario) K1A 0W9 Tél. : 613-941-2672
Téléc. : 613-954-1800 **www.irsc-cihr.gc.ca**

Canada

Institute of Aboriginal Peoples' Health
Institute of Aging
Institute of Cancer Research
Institute of Circulatory and Respiratory Health
Institute of Gender and Health
Institute of Genetics
Institute of Health Services and Policy Research
Institute of Human Development and Child and Youth Health
Institute of Infection and Immunity
Institute of Musculoskeletal Health and Arthritis
Institute of Neurosciences, Mental Health and Addiction
Institute of Nutrition, Metabolism and Diabetes
Institute of Population and Public Health

Institut de la santé des Autochtones
Institut du vieillissement
Institut du cancer
Institut de la santé circulatoire et respiratoire
Institut de la santé des femmes et des hommes
Institut de génétique
Institut des services et des politiques de la santé
Institut du développement et de la santé des enfants et des adolescents
Institut des maladies infectieuses et immunitaires
Institut de l'appareil locomoteur et de l'arthrite
Institut des neurosciences, de la santé mentale et des toxicomanies
Institut de la nutrition, du métabolisme et du diabète
Institut de la santé publique et des populations

March 21, 2013

Dr. Rachel Rosa ENGLER-STRINGER
Community Health and Epidemiology
College of Medicine
University of Saskatchewan
107 Wiggins Road
Saskatoon, Saskatchewan S7N 5E5

Dear Dr. ENGLER-STRINGER:

Congratulations on your success in the recent Canadian Institutes of Health Research funding competition.

You should take great pride in your success, particularly in light of the very competitive nature of CIHR peer review.

As you know, peer review is the cornerstone of our research funding system. This process rests on the kind voluntarism of your colleagues at other institutions who generously gave their time to review your application.

The Canadian Institutes of Health Research is committed to building an innovative national health research enterprise. To this end we have initiated a process to design a new Open Suite of Programs and peer review system to ensure the long-term sustainability of CIHR's contribution to the Canadian health research enterprise, to remove barriers, and to enable researchers from all pillars to improve CIHR's ability to deliver on its mandate.

To meet CIHR goals, we must also continue to communicate the value of health research to Canadians. That is why we encourage you to work with your institution to promote your research . We have developed guidelines on public communication, available on our website at http://www.cihr-irsc.gc.ca/e/30789.html, to support you in this activity.

Once again, congratulations and I wish you success in your research.

Yours sincerely,

Alain Beaudet, MD, Ph.D.
President

President
Canadian Institutes of Health Research
Room 97, 160 Elgin Steet, Address locator: 4809A
Ottawa, (Ontario) K1A 0W9 Tel.: (613) 941-2672
Fax (613) 954-1800 **www.cihr-irsc.gc.ca**

Président
Instituts de recherche en santé du Canada
Pièce 97, 160 rue Elgin, Indice de l'adresse: 4809A
Ottawa, (Ontario) K1A 0W9 Tél.: (613) 941-2672
Fax (613) 954-1800 **www.irsc-cihr.gc.ca**

Canada

Review Type/Type d'évaluation: Committee Member 1/Membre de comité 1
Name of Applicant/Nom du chercheur: ENGLER-STRINGER, Rachel Rosa
Application No./Numéro de demande: 297716
Agency/Agence: CIHR/IRSC
Competition/Concours: 2012-12-14 Operating Grant: Population Health Intervention Research to Promote Health and Health Equity/Subvention de fonctionnement : recherche interventionnelle en santé des populations visant à promouvoir la santé et l'équité en santé
Committee/Comité: Operating Grant: Population Health Intervention Research/Subvention de fonctionnement: Recherche interventionnelle en santé des populations
Title/Titre: The Good Food Junction: A Community-Based Food Intervention to Reduce Nutritional Health Inequities

---

Assessment/Évaluation:

The Good Food Junction: A Community Based Food Intervention to address Nutritional Health Adequacies is an evaluation of an intervention project called the “Good Food Junction”, a grocery store/health center developed to provide healthy food options in a Saskatoon food desert. The proposal seeks to evaluate the impact of the store on residents in surrounding neighbourhoods in order to better understand if and how interventions such as the one under study can lead to increased availability and purchasing of healthy food among both residents in the areas and users of the store specifically, and improved health related outcomes, in general and among vulnerable populations.

To do this, the researchers propose a cross-sectional survey of 600 households sampled door to door in the selected neighbours, a longitudinal study of 300 households drawn from the survey followed up at 6,12,and 18 months. The longitudinal survey will include measures from the CCHS, as well as specific questions on grocery shopping. Food purchasing data collected from GFJ will also be tracked over time. The food tracking data will also be used to evaluate a number of in-store interventions funded elsewhere.

A balanced team with experts in epidemiology, survey methods, nutrition, food programs, aboriginal health who have generally had experience working together. There is evidence of strong partnerships with community and provincial decision makers. Similarly the cooperation between the good food junction, the Station 20 West Centre, and other partners is well established and active. The PI team is a mixed of an early career researcher with a more established investigator. Clearly, the investigators are strong supporters of the intervention and programming and may benefit from voices outside of Saskatoon who may be less intimately involved. Otherwise, the environment is a supportive one for this research.

The issue of food deserts in communities is an important one and many communities are interested in addressing the issues of food security and healthy food availability. Systematic assessment and evaluation of community level programming is also more rare than it should be, particularly in the attempt to link programming and intervention to individual health outcomes. This proposal aims to provide information that will assess the impact of the specific program but should help other communities in the implementation of food availability interventions. Furthermore, most work has taken place outside of Canada. The work suggested here is well justified as important and novel.

Background literature supports the need for the study. The review is broadly supportive of an effect;

Review Type/Type d'évaluation: Committee Member 1/Membre de comité 1
Name of Applicant/Nom du chercheur: ENGLER-STRINGER, Rachel Rosa
Application No./Numéro de demande: 297716
Agency/Agence: CIHR/IRSC
Competition/Concours: 2012-12-14 Operating Grant: Population Health Intervention Research to Promote Health and Health Equity/Subvention de fonctionnement : recherche interventionnelle en santé des populations visant à promouvoir la santé et l'équité en santé
Committee/Comité: Operating Grant: Population Health Intervention Research/Subvention de fonctionnement: Recherche interventionnelle en santé des populations
Title/Titre: The Good Food Junction: A Community-Based Food Intervention to Reduce Nutritional Health Inequities

---

Assessment/Évaluation:

however, there is some question over the mechanism linking the availability of food to health outcomes. The nature of this mechanism (and the causal direction) is important for the development and implementation of interventions and it may be worth expanding on the potential for alternate explanations for the association between SES and obesity in particular, and provide further confidence that the intervention would have a causal effect. It should be considered that the potential reason for the lack of effect of previous interventions may be due to a lack of causal effect.

Research questions are in general clear, tied to the conceptual model, and well stated. Research question 4 is somewhat problematic. It is not immediately evident what the difference might be between a health impact and an early health impact. It is important to study the health impacts of these interventions, however, as stated in the baseline report, the effect of the intervention on health may be hard to demonstrate for many years and difficult to tie to a single intervention. While assumptions may be made about intermediate variables (such as additional purchasing of fruits and vegetables), answering research question 4 as part of the study's primary objectives may be challenging.

The piggybacking of intervention studies demonstrates the importance of the research and the value added by this study. However, it would be helpful to understand the specifics of the types of interventions that might be implemented and evaluated here.

This proposal has a number of strengths in terms of the methodology used. The existing baseline data assessment already completely prior to the intervention allows for pre-post comparisons, and demonstrates the ability of the research team. The use of administrative (food purchasing data) to assess changes in purchasing patterns appears to be a rich source of information. The longitudinal design would be effective at assessing changes in behaviour, purchasing, and health outcomes.

A timeline for the research would be helpful. The text suggests that the first wave of the survey will be conducted within 6 to 8 month from the opening of GFJ in September 2012, which suggests that the study will be conducted almost immediately upon the start of the study. Time for recruitment of neighbourhood based research assistants, or development and pre-testing of the survey does not appear to be included.

Review Type/Type d'évaluation: Committee Member 1/Membre de comité 1
Name of Applicant/Nom du chercheur: ENGLER-STRINGER, Rachel Rosa
Application No./Numéro de demande: 297716
Agency/Agence: CIHR/IRSC
Competition/Concours: 2012-12-14 Operating Grant: Population Health Intervention Research to Promote Health and Health Equity/Subvention de fonctionnement : recherche interventionnelle en santé des populations visant à promouvoir la santé et l'équité en santé
Committee/Comité: Operating Grant: Population Health Intervention Research/Subvention de fonctionnement: Recherche interventionnelle en santé des populations
Title/Titre: The Good Food Junction: A Community-Based Food Intervention to Reduce Nutritional Health Inequities

---

Assessment/Évaluation:

Sampling for the cross-sectional survey follows a study previously conducted in Toronto using residents of the neighbourhood to increase participation rates. The unit of sampling, randomly generated from an electoral list, appears to be a "household." Selection of participants is not unfortunately described. Are measures being collected for every member of the household or just the person who answers the door/person with the next birthday or other method for selecting participants within a household? Similarly, there do not appear to be inclusion or exclusion criteria for participants. For instance, are children and adolescents eligible to participate? As the measures for the longitudinal survey, in particular, are focused at the individual level (such as measured height and weight) rather than the household level.

Sampling will not be based on those who shop at GFJ. However, a stratified random sample would appear to have a number of advantages over a simple random sample. The sample size calculation suggest that half of the households would shop (regularly? ever?) at GFJ, but this figure is not justified in the text. Although the baseline survey in the appendix suggests that 55% would considering shopping regularly, there is a significant possibility that this figure would be lower—particularly if the intervention were less successful. A stratified sample would allow for sufficient sample size of exposed group for this study. Research question 4 moreover suggests stratifying the analysis by vulnerable population group, and sample size calculation should allow for this strata to be sufficiently large to provide useful data.

In the "nested" longitudinal design, a subset of the households are chosen for inclusion but the process for selecting households from the cross-sectional survey is not described. Nor whether the selection process (and/or consent to for follow up) takes place during the initial recruitment after the initial analysis of the cross-sectional data.

The analysis of the use of the food purchasing data (fixed and random effects models) to answer research question two suggests that individual level data will be available. However, this appears to assume that postal codes will uniquely identify individuals and the validity of this assumption needs to be justified. It would be useful to assess the food purchasing habits of the longitudinal sample but this is not described. Changes in food purchasing are likely to be confounded with other effects such as prices and season but it isn't clear if these are to be controlled for in the analysis.

**Review Type/Type d'évaluation:**	Committee Member 1/Membre de comité 1
**Name of Applicant/Nom du chercheur:**	ENGLER-STRINGER, Rachel Rosa
**Application No./Numéro de demande:**	297716
**Agency/Agence:**	CIHR/IRSC
**Competition/Concours:**	2012-12-14 Operating Grant: Population Health Intervention Research to Promote Health and Health Equity/Subvention de fonctionnement : recherche interventionnelle en santé des populations visant à promouvoir la santé et l'équité en santé
**Committee/Comité:**	Operating Grant: Population Health Intervention Research/Subvention de fonctionnement: Recherche interventionnelle en santé des populations
**Title/Titre:**	The Good Food Junction: A Community-Based Food Intervention to Reduce Nutritional Health Inequities

Assessment/Évaluation:

The KTE plan is vague; however, it appears that the researchers have considerable experience and interest in ensuring that research uptake occurs. The research team and collaborators also suggests an integrated knowledge exchange process that ensure excellent KTE within Saskatoon/Saskatchewan. Other efforts to expand the impact nationally and internationally would also be valuable.

**Review Type/Type d'évaluation:**	Committee Member 2/Membre de comité 2
**Name of Applicant/Nom du chercheur:**	ENGLER-STRINGER, Rachel Rosa
**Application No./Numéro de demande:**	297716
**Agency/Agence:**	CIHR/IRSC
**Competition/Concours:**	2012-12-14 Operating Grant: Population Health Intervention Research to Promote Health and Health Equity/Subvention de fonctionnement : recherche interventionnelle en santé des populations visant à promouvoir la santé et l'équité en santé
**Committee/Comité:**	Operating Grant: Population Health Intervention Research/Subvention de fonctionnement: Recherche interventionnelle en santé des populations
**Title/Titre:**	The Good Food Junction: A Community-Based Food Intervention to Reduce Nutritional Health Inequities

Assessment/Évaluation:

1. Research Approach

This proposal will investigate the impact of a newly established population health intervention (i.e. a local neighborhood full-service food store called the Good Food Junction) on the health of people in its surrounding neighborhoods. This will be accomplished by obtaining data from three studies (a cross-sectional household survey, a nested longitudinal study and a food-purchasing tracking study) to answer four research questions on : 1) awareness of the GFJ; 2) trends in food purchasing over time; 3) changes in food purchasing following a number of GFJ-initiated advertising promotions; and 4) impact of GFJ on selected health outcomes, in general, and for selected vulnerable groups.

With respect to the cross-sectional study, it does not appear that the selection of the households will, in fact, be random. The eligibility criteria for participating households has also not been specified. The sample size calculation is unclear and it is difficult to know the magnitude of the 'small effects' that is described. It is also difficult to understand how household food purchasing patterns will take into account household size and other socio-demographic characteristics which influence food purchasing. In assembling the study population, there does not seem to be consideration for how vulnerable groups (Aboriginal people, seniors and new immigrants) will be identified.

For the nested longitudinal study, there is no justification for the sample size of 300 (200 who purchase food at GFJ and 100 who don't). Also, how will the definitions of households who do purchase and who don't purchase food at GFJ be determined? For example, what if only one household member purchases at GFJ? From whom will the Food Frequency Module be obtained? Who will contribute the self-reported information on the health outcomes? Because the detail on methods is insufficient it is difficult to understand how the intended response to the research questions will be obtained.

For the food purchasing tracking study, how the data will be compared to the rest of Saskatoon is unclear. How a stratified analysis will be conducted between residents and non-residents is also unclear.

The strengths of the proposal include 1) its relevance and timeliness in terms of the topic and the recent introduction of a population heath intervention into a new environment ; 2) having the research assistants come from the local neighborhoods; 3) collaboration of the GFJ and local health authorities; 4) a strong

**Review Type/Type d'évaluation:**	Committee Member 2/Membre de comité 2
**Name of Applicant/Nom du chercheur:**	ENGLER-STRINGER, Rachel Rosa
**Application No./Numéro de demande:**	297716
**Agency/Agence:**	CIHR/IRSC
**Competition/Concours:**	2012-12-14 Operating Grant: Population Health Intervention Research to Promote Health and Health Equity/Subvention de fonctionnement : recherche interventionnelle en santé des populations visant à promouvoir la santé et l'équité en santé
**Committee/Comité:**	Operating Grant: Population Health Intervention Research/Subvention de fonctionnement: Recherche interventionnelle en santé des populations
**Title/Titre:**	The Good Food Junction: A Community-Based Food Intervention to Reduce Nutritional Health Inequities

Assessment/Évaluation:

research team; and 5) a combination of studies collecting different types of data to inform the overall impact of the intervention.

2. Applicants

There is a strong academic research team with expertise in nutrition and epidemiology. Several have collaborated together in the past and have made important contributions to research on food environments. The nominated PI joined the Department of Community Health and Epidemiology in 2009 as an Assistant Professor after completing postdoctoral training in 2008. Her previous work experience includes being a Research Coordinator on several nutrition projects undertaken by the Department of Pharmacy and Nutrition of the University of Saskatchewan and, for shorter periods of time, a lecturer, teaching assistant and instructor.

Dr. Muhajarine is the co-PI on this proposal. He is a Full Professor and Chair of the Department of Community Health and Epidemiology. He is a highly productive, well-funded and renowned scholar and has made important scientific contributions in the field of nutrition and health in different population groups.

These PIs are complemented by five other academic colleagues from their and other academic departments at the University of Saskatchewan, contributing appropriate expertise in health inequities, nutrition and environment.

3. Environment for the Research

There is sufficient past collaborative research experience to expect a supportive research environment at the University of Saskatchewan. In addition, this proposal continues pilot research on this topic by this group funded by CIHR. There are letters of support from Saskatoon Health and Saskatoon Community Services in addition to the larger environment in which Good Food Junction is situated (i.e. Station 20 West and CHEP). The Good Food Junction itself is a partner in this research.

Review Type/Type d'évaluation: Committee Member 2/Membre de comité 2
Name of Applicant/Nom du chercheur: ENGLER-STRINGER, Rachel Rosa
Application No./Numéro de demande: 297716
Agency/Agence: CIHR/IRSC
Competition/Concours: 2012-12-14 Operating Grant: Population Health Intervention Research to Promote Health and Health Equity/Subvention de fonctionnement : recherche interventionnelle en santé des populations visant à promouvoir la santé et l'équité en santé
Committee/Comité: Operating Grant: Population Health Intervention Research/Subvention de fonctionnement: Recherche interventionnelle en santé des populations
Title/Titre: The Good Food Junction: A Community-Based Food Intervention to Reduce Nutritional Health Inequities

---

Assessment/Évaluation:

4. Impact of the Research

It is clear that the introduction of a new source of good food into a previously 'desert area' will have an impact on food access and food consumption patterns. The ability of this research to make a direct link between the Good Food Junction and health outcomes is unclear, given that other similarly new interventions are also at play in the immediate surroundings which also affect these same outcomes (e.g. Food kitchens and mental health).

Review Type/Type d'évaluation: SO Notes /Notes de l'agent scientifique
Name of Applicant/Nom du chercheur: ENGLER-STRINGER, Rachel Rosa
Application No./Numéro de demande: 297716
Agency/Agence: CIHR/IRSC
Competition/Concours: 2012-12-14 Operating Grant: Population Health Intervention Research to Promote Health and Health Equity/Subvention de fonctionnement : recherche interventionnelle en santé des populations visant à promouvoir la santé et l'équité en santé
Committee/Comité: Operating Grant: Population Health Intervention Research/Subvention de fonctionnement: Recherche interventionnelle en santé des populations
Title/Titre: The Good Food Junction: A Community-Based Food Intervention to Reduce Nutritional Health Inequities

---

Assessment/Évaluation:

An ambitious project.

Strengths Important and timely question with few studies performed for an intervention that may be relevant to other setting. Measuring important and relevant outcomes.

Balanced and large team with good partnerships.

Combination of different approaches which adds important breadth of the proposal.

Weaknesses Sampling of the survey was the only major concern. This concern could be addressed, but reviewers had difficulty giving the proposal strong support as the described in the proposal. Sampling households? people within the household? Sample power was not clearly justified. It was challenging to see how the food purchases were to be connected to survey.

Causal relationship and/or separation of other interventions/programs was a challenge - but one that was recognized by the investigators.

It seemed unclear with seemingly high office expenses. That said, the proposal seemed ambitious for the funding that will be allocated.
